# Anomaly Detection Method for Harmonic Reducers with Only Healthy Data

**DOI:** 10.3390/s24237435

**Published:** 2024-11-21

**Authors:** Yuqing Li, Linghui Zhu, Minqiang Xu, Yunzhao Jia

**Affiliations:** Deep Space Exploration Research Center, Harbin Institute of Technology, Harbin 150001, China; bradley@hit.edu.cn (Y.L.); 20b918054@stu.hit.edu.cn (L.Z.); 17b918051@hit.edu.cn (Y.J.)

**Keywords:** anomaly detection, fault detection, harmonic reducer, auto-encoder (AE), one-class support vector machine (OCSVM)

## Abstract

A harmonic reducer is an important component of industrial robots. In practical applications, it is difficult to obtain enough anomaly data from error cases for the supervised training of models. Whether the information contained in regular features is sensitive to anomaly detection is unknown. In this paper, we propose an anomaly detection frame for a harmonic reducer with only healthy data. We considered an auto-encoder trained using only healthy features, such as feature mapping, in which the difference between the output and the input constitutes a new high-dimensional feature space that retained information relevant only to anomalies. Compared to the original feature space, this space was more sensitive to abnormal data. The mapped features were then fed into the OCSVM to preserve the feature details of the abnormal information. The effectiveness of this method was validated by multiple sets of data collecting from harmonic reducers. Three different residual calculations and four different AE models were used, showing that the method outperforms an AE or an OCSVM alone. It is also verified that the method outperforms other typical anomaly detection methods.

## 1. Introduction

Anomaly detection is a key step in monitoring the condition of a machine. It can determine whether more effort needs to be allocated to fault diagnosis or maintenance, thus increasing the remaining life of the machine and reducing costs. Currently, most of the anomaly detection methods for machinery and equipment involve signal analysis (vibration signal analysis [1], acoustic emission signal analysis [2]) and require pre-existing knowledge for comparison (e.g., pre-set thresholds, frequency of specific faults.). These methods require corresponding expert experience and the a priori knowledge of machines and their operating conditions.

As an important component of industrial robots, the reliability study of harmonic reducers is an essential part of guaranteeing the production link of industrial robots. Hu [3] et al. proposed an accuracy performance prediction method based on transfer learning and Gaussian process regression (GPR) to solve the performance prediction problem of harmonic reducers. Zhang [4] et al. proposed a method using optimal variational mode decomposition (VMD) to extract harmonic reducer misalignment fault features. Wu [5] et al. proposed a new method to construct a health index using motor current signals and vibration signals, combining multiple signal processing methods to obtain multiple features and eliminating the redundant features among them for the life prediction of harmonic reducers.

However, all of the above methods require expert experience and a priori knowledge. In order to overcome these limitations, there is an increasing tendency to use machine learning methods for fault detection [6]. Jia [7] et al. designed a framework for identifying the dynamic failure modes of a harmonic reducer, and used Hidden Markov Models to extract the new failure features, which can effectively identify the failure modes of the harmonic reducer under the dynamically changing working conditions. Zhi [8] et al. utilized the acoustic emission signals to realize the fault detection of the harmonic reducer; they also used the CNN-LSTM model, combined with a WRCTD algorithm, to realize the fault detection of the acoustic emission signal of a harmonic reducer [9]. At present, the research group for the harmonic reducer is still focusing on improving its basic performance. The research on the reliability of harmonic reducers mainly focuses on the field of fault identification, which requires the collection of a certain number of samples with different kinds of faults.

In practice, the collection of fault data is often accompanied by high equipment costs. At the same time, there may be unknown faults that make the fault identification method ineffective. Anomaly detection methods, which are trained only on normal data, have become a hot topic of research in recent years [10]. Commonly used anomaly detection methods can be broadly categorized as statistical-based [11], model-based [12,13,14], deep learning-based [15], etc. Patil, A et al. [16] summarized the commonly used data-driven anomaly detection methods in industrial systems, elaborated on the anomaly detection methods for different industrial components (bearings and gears) and industrial equipment (gas turbines, wind turbines, thermal power plants, etc.), and summarized the advantages and disadvantages of different methods. Staerman G et al. [17] compared the advantages and disadvantages of several commonly used anomaly detection methods using helicopter data and building spectral data.

An auto-encoder (AE) is an artificial neural network trained on raw or processed data in an unsupervised manner. Since it only needs to be trained on healthy data, it is naturally suited to deal with the anomaly detection problem under the condition of a lack of anomaly data. Ali Nawaz et al. [18] summarized the application of an ensemble of AEs in the field of biological anomaly detection, and evaluated the advantages and disadvantages of different AE combinations. Reference [19] summarizes the AE-based anomaly detection method for industrial processes. Jiang et al. [20] proposed an anomaly detection method combining spatio-temporal features by using a Stack spatial–temporal auto-encoder to reconstruct the signal to reduce the effect of anomalous signals. Windmann S et al. [21] proposed a hybrid model of a regularized LSTM and an AE for fault detection in automated systems. Zhu et al. [22] used a VAE for the fault monitoring of industrial production processes and proposed a fault detection framework.

However, the use of an AE alone for anomaly detection may result in the high reconstruction ability of anomaly data. In order to improve the detection rate of anomaly data, people have not only improved AE-based anomaly detection methods, such as the use of inpainting [23], but have also investigated anomaly detection methods by combining an AE with other models [24,25,26].

The OCSVM, the most robust unsupervised outlier detection method, has been successfully applied in the field of anomaly detection [27,28,29]. To date, there have been several studies combining AEs with OCSVMs for fault detection. In [30], gas pipeline leakage was effectively detected by using an improved LSTM-AE to extract features from the natural gas pipeline data and an OCSVM for decision making; Ref. [31] combined a VAE and an OCSVM to realize anomaly detection in industrial control networks; and [32] used an improved stack-AE for feature extraction, and an SVM for decision making to realize anomaly detection and fault classification.

Most of the above works use an AE as a feature extraction tool to compress the original signal into a lower dimensional, sparser space. The unknown harmonic reducer anomaly frequency may be high, thus requiring a high sampling frequency. Using an AE as a feature extraction tool requires significant training and computational costs. In contrast to them, we use conventional time domain features and frequency domain features to form the initial training dataset. After mapping via AE, the original feature space is transformed into a new space that is more sensitive to faults. The mapped features are then fed into the OCSVM model trained from the health data for decision making, preserving the details about the anomalies in the features. The main contributions of this paper are as follows:

1. A new anomaly detection frame using only normal data for harmonic reducers was proposed. This method addressed the problems of a lack of anomaly data and unknown failure modes in anomaly detection, and only required training on healthy data;

2. Unlike most AE-based anomaly detection methods, the AE in this paper does not act as a feature extractor, but as a feature mapping tool. The vibration signals were collected at a high frequency, and training AE directly with the raw signals would consumed a lot of training time, and the generalized signal features may not have accurately reflected the difference between the normal and abnormal signals. By removing the health information from the features after AE mapping, the abnormality sensitivity of the model was improved;

3. Using OCSVM instead of thresholding for anomaly decision making retained more anomaly information and further improved the model’s sensitivity to anomalies.

This paper is organized as follows: Section 2 introduces the related preparatory knowledge, Section 3 describes the specific structure of the anomaly detection method for the harmonic reducer proposed, Section 4 describes the experiments for harmonic reducer data acquisition, Section 5 describes the results and discussion, and Section 6 is the conclusion.

## 2. Preliminary

In this section, the preparatory knowledge of the AE-OCSVM-based harmonic reducer anomaly detection framework is presented. This includes the basic knowledge of the different auto-encoders used in this study and the basic knowledge of the OCSVM.

### 2.1. Auto-Encoder (AE)

An auto-encoder (AE) is a type of algorithm with the primary purpose of learning an “informative” representation of data that can be used for different applications by learning to reconstruct a set of input observations well enough [33]. It can be simply understood as a neural network with the same inputs and learning objectives, and its structure is divided into two parts: encoder and decoder. It can be written as the following function:

(1) $$\tilde{X_{i}}=f(h_{i})=f(g(X_{i}))$$

where $\tilde{X_{i}}\in {\mathbb{R}}^{n}$ is the output of the decoder f, $X_{i}\in {\mathbb{R}}^{n}$ is the input, an $h_{i}\in {\mathbb{R}}^{q}$ (the latent feature representation) is the output of the encoder g. Training an AE can be simply understood as finding g, f that satisfies

(2) $$\arg\underset{f,g}{\min}<[\Delta (X_{i},f(g(X_{i})))]>$$

where Δ indicates the measure of difference between the input and the output and <⋅> indicates the average of all the observations. Similarly to neural networks, the layers of the AE need to be connected to each other by activation function, which introduce a nonlinear representation, improve the expressive power of the network, and solve problems that cannot be solved by linear models. The ReLU activation function has good performance for accelerating model training while reducing the risk of overfitting [34]:

(3) ReLU(x)=max(0,x)

Similarly to neural networks, the goal of training is to minimize the loss function:

(4) $$L_{\mathrm{MSE}}=MSE=\frac{1}{M}\sum_{i=1}^{M}|X_{i}-\tilde{X_{i}}{|}^{2}$$

where M indicates the number of samples. It is easy to see that the $L_{MSE}$ achieves the minimum value when the input is equal to the output. Typically, the *MSE* is also used as the reconstruction error of the AE. For the anomaly detection problem, since the AE is trained from normal samples, its reconstruction ability for anomalous data is worse than that of normal data, and the reconstruction error becomes larger. When the reconstruction error is greater than a certain threshold, the data are considered abnormal.

**Stacked auto-encoder (StAE)**

A stacked auto-encoder is also called a deep auto-encoder, which takes the output of the previous layer of an encoder/decoder as the input to the next layer. Assuming that an n−m−k stacked auto-encoder is trained, it is necessary to compute the n−m−n encoding and decoding transforms, then the m−k−m encoding and decoding transforms, and finally stack these transforms together.

**Sparse auto-encoder (SpAE)**

The sparse auto-encoder introduces a sparsity penalty term that makes the neuron activations in the hidden layer sparser, which is used to better capture the important features of the data [35]. In this paper, L1 regular terms are used to add sparsity penalty terms; the training object is rewritten as follows:

(5) $$\min L_{MSE}+\lambda \sum_{i=1}^{M}\left|w_{i}\right|$$

where w is the weight of the sparse auto-encoder.

**Variational auto-encoder (VAE)**

VAE is a deep generative network based on variable Bayes inference proposed by Kingma [36] in 2013. The goal of the inference network (encoder) is to make the distribution $q_{\varphi }(z|x)$ as close as possible to the true posterior distribution $p_{\theta }(z|x)$, where φ, θ are their parameters, respectively. That is, a set of parameters $\varphi^{*}$ needs to be solved to minimize the KL divergence:

(6) $$\varphi^{*}=\underset{\varphi }{\arg\min}\;KL(q_{\varphi }(z|x),p_{\theta }(z|x))$$

The maximum likelihood function for given x is

(7) $$\begin{array}{l} \log p_{\theta }(x|z)=\int_{z}q_{\varphi }(z|x)\log(\frac{p_{\theta }(x|z)p_{\theta }(z)}{q_{\varphi }(z|x)})dz-\int_{z}\frac{p_{\theta }(z|x)}{q_{\varphi }(z|x)}q_{\varphi }(z|x)dz\\ =ELBO+KL(q_{\varphi }(z|x),p_{\theta }(x|z)) \end{array}$$

where ELBO means Evidence Lower Bound. Therefore, the objective function changes from $\underset{\varphi }{\arg\min}KL(q_{\varphi }(z|x),p_{\theta }(z|x))$ to $\underset{\varphi }{\arg\max}ELBO$.

Similarly, the goal of a generation network is to solve a set of network parameters $\theta^{*}$ to maximize the ELBO. The final objective function of the VAE is

(8) $$\begin{array}{l} \underset{\theta ,\varphi }{\max}ELBO=\underset{\theta ,\varphi }{\max}\int_{z}q_{\varphi }(z|x)\log(\frac{p_{\theta }(x|z)p_{\theta }(z)}{q_{\varphi }(z|x)})dz\\ =\underset{\theta ,\varphi }{\max}\int_{z}\log p_{\theta }(x|z)q_{\varphi }(z|x)dz-\int_{z}\log(\frac{q_{\varphi }(z|x)}{p_{\theta }(z)})q_{\varphi }(z|x)dz\\ =\underset{\theta ,\varphi }{\max}{Ε}_{z\sim q_{\varphi }(z|x)}[\log p_{\theta }(x|z)]-KL(q_{\varphi }(z|x)||p_{\theta }(z)) \end{array}$$

The first term is the reconstruction loss, and the second term is the KL divergence.

**Long Short-Term Memory Auto-encoder**

**Figure 1:**
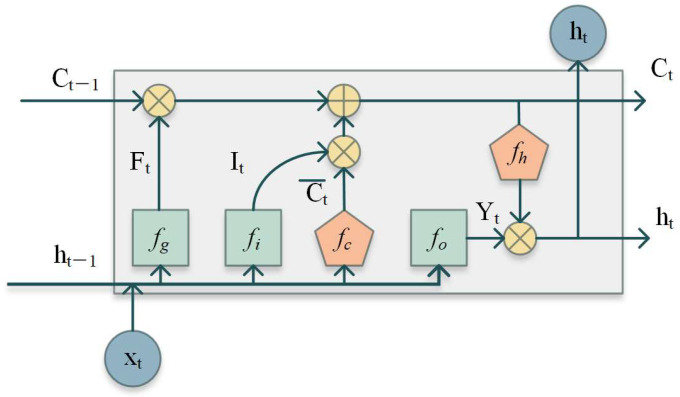
Unit of LSTM.

The encoding and decoding process of the LSTM-AE is performed by the LSTM [Figure 1] network. It effectively overcomes the problem of vanishing or exploding gradients and better retains valid information [37].

First, the LSTM generates decision vectors (the optimization variable) and selects candidate information:

(9) $$I_{t}=f_{i}(w_{i}x_{t}+w_{i}h_{t-1}+b_{i})$$

where $w_{i}$, and $b_{i}$ are the weight and bias between two components, respectively. h is the hidden state. x is the input.

(10) $$F_{t}=f_{g}(w_{g}x_{t}+w_{g}h_{t-1}+b_{g})$$

where $F_{t}$ is the vector of forget gate. $w_{g}$ and $b_{g}$ are the weight and bias of the forget gate, respectively. Then, the input candidate information $\tilde{C_{t}}$ is generated as follows:

(11) $$\tilde{C_{t}}=f_{c}(w_{c}x_{t}+w_{c}h_{t-1}+b_{c})$$

The current state $C_{t}$ is

(12) $$C_{t}=C_{t-1}F_{t}+{\tilde{C}}_{t}+I_{t}$$

The output is

(13) $$\begin{array}{l} Y_{t}=f_{o}(w_{o}x_{t}+w_{o}h_{t-1}+b_{o})\\ h_{t}=Y_{t}f_{h}(C_{t}) \end{array}$$

### 2.2. One-Class Support Vector Machine (OCSVM)

The OCSVM is one of the most commonly used one-class classifiers. It takes the origin of the feature space as an outlier and obtains the optimal hyperplane by maximizing the distance between the origin and the target. This optimization problem can be described as follows:

(14) $$\begin{array}{l} \underset{w,\rho ,\xi }{\min}\frac{1}{2}{\left\|w\right\|}^{2}-\rho +\frac{1}{\nu N}\sum_{i=1}^{N}\xi_{i}\\ s.t.w^{T}\Phi (x_{i})\geq \rho -\xi_{i}\\ \xi_{i}\geq 0,i=1,2,\ldots ,N \end{array}$$

where ξ is the vector of slack variables, w is the hyperplane normal vector, Φ is the kernel function, $\left\|\cdot \right\|$ is the $l_{2}$-norm, ν∈(0,1] is the trade-off parameter that represents the upper bound on the fraction of the training samples lying on the wrong side of the hyperplane and the lower bound on the fraction of support vectors, and N is the number of training samples. According to the Lagrange multiplier method, the equation can be written as follows:

(15) $$\begin{array}{l} \underset{\alpha }{\min}\frac{1}{2}\sum_{i=1}^{N}\sum_{j=1}^{N}\alpha_{i}\alpha_{j}K(x_{i},x_{j})\\ s.t.\sum_{i=1}^{N}\alpha_{i}=1\\ 0\leq \alpha_{i}\leq \frac{1}{\nu N},i=1,2,\ldots ,N \end{array}$$

α is a Lagrange multiplier. K(⋅) is the kernel equation satisfying $K(x,y)=\Phi {\left(x\right)}^{T}\Phi (y)$. The normal vector w and bias term ρ can be written as follows:

(16) $$\begin{array}{l} w=\sum_{i=1}^{N}\alpha_{i}\Phi (x_{i})\\ \rho =\sum_{j=1}^{N}\alpha_{j}K(x_{j},x_{i}) \end{array}$$

Ultimately, the decision equation with kernel expansion and the class label can be written as follows:

(17) $$\begin{array}{l} f(x)=\sum_{i=1}^{N}\alpha_{i}K(x_{i},x)-\rho \\ \hat{y}=sign(f(x)) \end{array}$$

## 3. Method

This section presents our proposed anomaly detection framework for harmonic reducers.

### 3.1. Anomaly Detection Framework for Harmonic Reducers

The AE is naturally suited to handling anomaly detection problems, but its ability to detect anomalies is limited by the fact that its reconstruction of anomalous data may also be accurate. Therefore, in this study, we only use the AE as a feature mapping tool. Since there is only normal data in the training data, the reconstructed residual features can be understood as the performance of the features after removing the “normal components”. Then, we chose an OCSVM, a one-class classifier suitable for multidimensional feature anomaly detection, to learn the new normal features.

**Figure 2:**
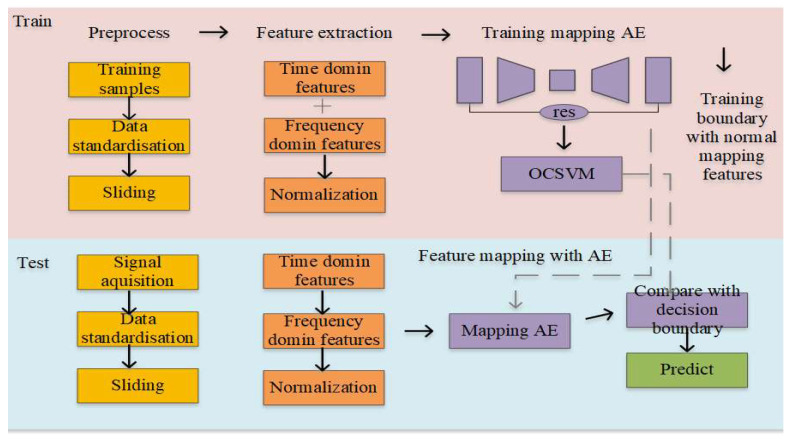
Structure of AE-OCSVM model.

The harmonic reducer anomaly detection framework proposed in this paper can be divided into four parts: data acquisition and preprocessing, feature extraction, feature mapping, and the decision with the OCSVM boundary, as shown in Figure 2.

### 3.2. Data Acquisition and Preprocessing

The experimental part of the data acquisition will be described specifically in Section 4. We needed to standardize the collected data first so that the data as a whole conformed to a Gaussian distribution:

(18) $$x_{new}=\frac{x-\mu }{\sigma }$$

where μ is the mean of x, σ is the standard deviation of x.

### 3.3. Feature Extraction

The time cost of training with raw data is very high; we performed feature extraction on the original data to improve the computational efficiency. Considering the lack of expert experience with harmonic reducer failure modes, we extracted some commonly used time domain features and frequency domain features; the specific features are shown in Table 1. In order to eliminate the effect of the magnitude between the features, we needed to normalize the features so that the distributions of features of the different magnitudes all fell between 0 and 1.

### 3.4. Feature Mapping with AE

Since the AE was trained using only normal data, it could not fit the anomalous data well, so there was a difference between the residual vectors of the normal and anomalous data after mapping with the AE. From the perspective of feature mapping, an AE trained with normal data can be viewed as a mapping function $f_{AE}(x)=res(x,\tilde{x})$; x is the input, $\tilde{x}$ is the reconstruction of x, and res(⋅) denotes the difference between the reconstructed feature and the original feature. $f_{AE}$ maps the original feature space to another “difference space”. Typically, AE uses $MSE={\sum_{i=1}^{M}\left|x_{i}-{\tilde{x}}_{i}\right|}^{2}/M$ as the indicator for anomaly detection, which means when $res=x-\tilde{x}$, we consider that there is a difference in the average distance to the origin between the normal and abnormal data in the “difference space”. In this paper, we validated three different kinds of res:

(19) $$\begin{array}{l} res1=x-\tilde{x}\\ res2=\left|x\right|-\left|\tilde{x}\right|\\ res3=x^{2}-{\tilde{x}}^{2} \end{array}$$

where x denotes each element of the input, $\tilde{x}$ denotes each element of the reconstruction.

### 3.5. Decision with OCSVM

Compared to using a single MSE as a decision value, directly using the multidimensional mapped features as classification features can better preserve the details of the difference. We input the AE-mapped features of the training samples into the OCSVM for training boundary. The trained boundaries were then used for testing.

## 4. Experiment

We conducted several sets of harmonic reducer experiments to verify the proposed framework, which are outlined in this section. Also, in order to verify the effect of different AE on the anomaly detection results of the harmonic reducer, four different kinds of AE with similar structures were selected.

### 4.1. Experimental Setup

**Figure 3:**
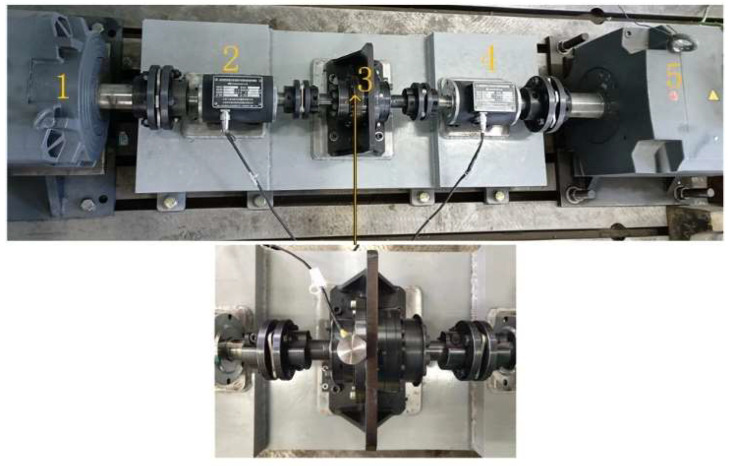
Harmonic reducer test bench.

In this experiment, the model of the harmonic reducer used was LCSG-32-50-C-I (leaderdrive, Suzhou, China), the ratio of this harmonic reducer was 50, the maximum speed was 4500 rpm, and the transmission gap was 10 arcmin. In order to simulate the real working conditions of the harmonic reducer in the real application environments, we designed a test bench that could provide speed variation and torque variation. Figure 3 shows the harmonic reducer test bench: 1 is the drive motor providing speed of model 1PH8163 44 kW Siemens, 2 is the speed sensor detecting the input speed, 3 is the harmonic reducer for the test, 4 is the speed sensor detecting the output speed, and 5 is the loading motor providing torque for model 1PH8137 22 kW Siemens. The acquisition system was a DEWETRON M7 system. An acceleration sensor with a bandwidth ranging from 1 Hz to 4000 Hz was used to acquire acceleration vibration signals. The sampling frequency was 100 kHz.

**Figure 4:**
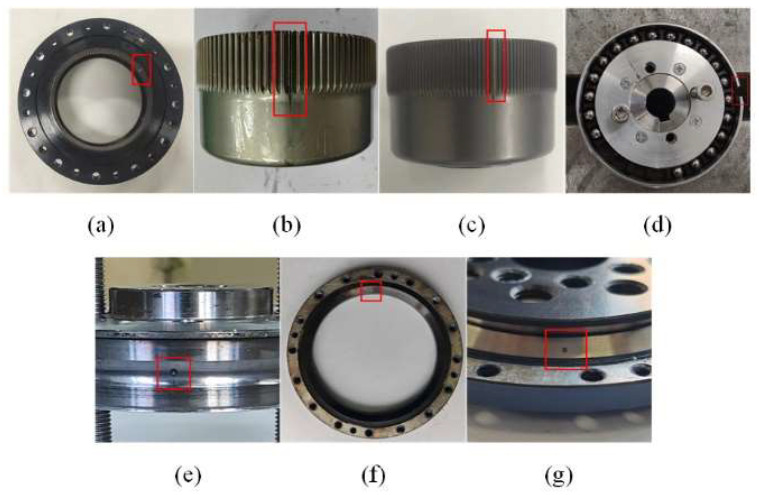
Ref. [7] faulty part: (**a**) circular spline gear crack fault; (**b**) flex–spline gear wear fault; (**c**) flex–spline gear crack fault; (**d**) flexible bearing outer race semi-crack fault; (**e**) flexible bearing inner race wear; (**f**) cross roller bearing outer race fault; (**g**) cross roller bearing inner race fault.

In practical applications, gears and bearings are subjected to alternating stresses for long periods of time, often resulting in cracks or fractures. Inadequate lubrication or the presence of external particles can lead to wear problems. In order to simulate the anomalies that may occur in harmonic reducers in real application scenarios, there were seven types of harmonic reducer anomalies tested in this experiment [Figure 4], which are as follows: (a) F1—circular spline gear crack fault, wire-cut machining, wear of 0.4 mm; (b) F2—flex–spline gear wear fault, electrical discharge machining (EDM), wear of 0.2 mm; (c) F3—flex–spline gear crack fault, wire-cut machining, wear of 0.4 mm; (d) F4—flexible bearing outer race semi-crack fault, wire-cut machining, wear of 0.2 mm; (e) F5—flexible bearing inner race wear, EDM, pitting diameter of 1.2 mm; (f) F6—cross roller bearing outer race fault, EDM, pitting diameter of 1.2 mm; (g) F7—cross roller bearing inner race fault, EDM, pitting diameter of 1.2 mm. When performing a fault test, we will replace the corresponding faulty part, setting only one fault at a time, while the other parts are healthy.

**Figure 5:**
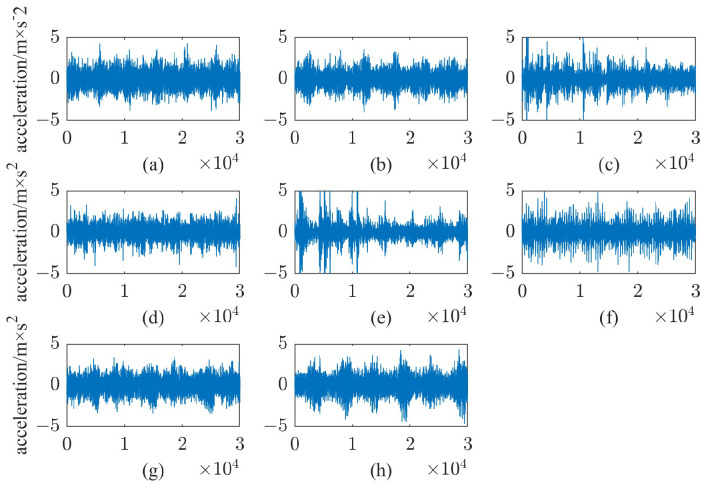
Vibration signal after standardization: (**a**) normal; (**b**–**h**): F1–F7.

In order to simulate the different working conditions of the harmonic reducer in practical applications, six different rotational speeds were set in this experiment, and three torques were set under each rotational speed, and the specific experimental parameters are shown in Table 2. The length of each sample was 30,000. The original vibration signals of the harmonic reducer after standardization are shown in Figure 5.

### 4.2. Parameters

**Figure 6:**
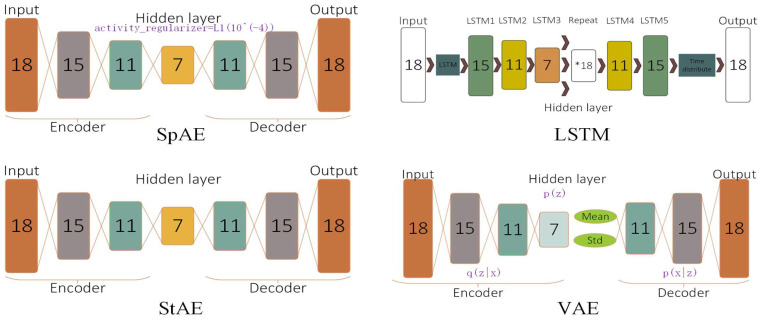
Structure of AE.

In order to verify the effect of different AEs on the experimental results, we chose four different AEs, including a stacked auto-encoder (StAE), a sparse auto-encoder (SpAE), a variational auto-encoder (VAE), and an LSTM auto-encoder (LSTM). We tried to ensure that these encoders had similar structures and observed their anomaly detection results; the specific structures of the encoders are shown in Figure 6. Since the cost of misclassifying anomalies is much higher than misclassifying normal data, we wanted to tighten the thresholds and boundaries to reduce the possibility of misclassifying the anomalies, and therefore the threshold was set to the first 95% of the training MSE.

When training AE, we set the maximum number of epochs to 100, and in order to prevent overfitting, we set an early stopping: when the loss decreased less than 0.0001, patience = 5. We set the kernel function of OCSVM as RBF with the degree = 3. To improve the ability of anomaly detection, we divided the 5% training samples nearest to the origin as the anomaly samples for training.

### 4.3. Evaluation Indicators

We chose different evaluation metrics for assessing the performance of the model: the accuracy (ACC), the F1 score, and the Area Under Curve (AUC). At the same time, considering the impact of sample imbalance on the calculation of accuracy, we calculated the accuracy of the test samples with the real labels of “normal” and “abnormal”, respectively, and calculated the mean value.

### 4.4. Baseline

In order to compare the anomaly detection performance of the proposed method with the AE and the OCSVM, we first compared the results with the AE only and the OCSVM only. Secondly, the best model was selected and compared to other anomaly detection methods:

XGBoost Regressor [38] (contamination = 0.05): An integrated learning algorithm based on gradient boosting decision trees. Its core idea is to work together on a learning task by constructing multiple weak learners (decision trees in this paper) and combining them in a weighted way;

GAAL (Mo-GAAL, So-GAAL) [39] (contamination = 0.05, max epoch = 100, k = 10): generative adversarial active learning is used to generate informative outliers directly, which solves the problem of missing information caused by “dimensionality catastrophe”;

Bagging (AvgBag) [40] (contamination = 0.05): The Bagging method reduces generalization error by combining several models. The main idea is to train several different models separately and then have all the models vote on the output of the test samples. In this paper, several decision tree models were used to train the model for each resampled sample and average the output;

AvgKNN [41] (contamination = 0.05): AvgKNN uses the average distance to k’s nearest neighbors as the outlier score;

Elliptic Envelope (EE) (contamination = 0.05): An Elliptic Envelope assumes that the data are normally distributed. Based on this assumption, an ellipse is “drawn” around the data, classifying any sample inside the ellipse as normal and any sample outside the ellipse as anomalous;

Isolation Forest [42] (IF): An Isolation Forest considers anomalous samples to be sparsely distributed and far away from high-density populations. An anomalous samples can be isolated by a fewer number of random feature segmentations compared to the normal samples;

Random Forest Regressor [43] (RF): The Random Forest Regressor utilizes the integration of multiple decision trees to perform regression calculations and average the final results;

Local Outlier Factor (LOF) [44]: The LOF is a density-based outlier detection algorithm. This method assigns an outlier factor, LOF, to each sample that depends on the neighborhood density, which determines whether the sample is an outlier or not.

## 5. Result and Discussion

### 5.1. Results for Different Mapping Features

**Figure 7:**
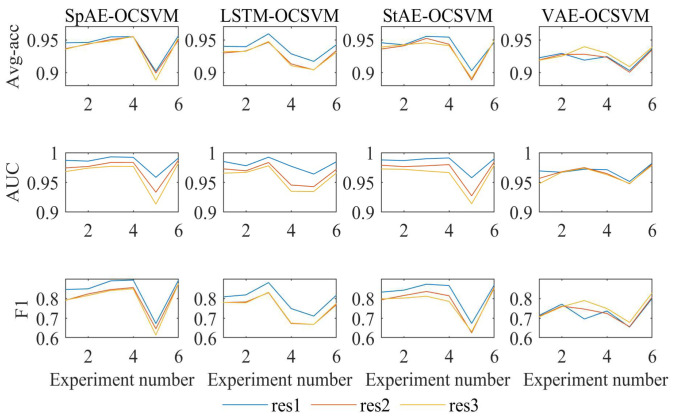
Results with different res.

We computed the results for three kinds of mapping features (*res*), as shown in Figure 7.

**Avg-acc**

The Avg-acc was greater than 90% for all the working conditions when using res1. Each model reached its lowest Avg-acc in Experiment 5. For both SpAE-OCSVM and StAE-OCSVM, the Avg-acc for Experiments 1, 2, 3, 4, and 6 were around 95%. For the SpAE-OCSVM, the LSTM-OCSVM, and the StAE-OCSVM, the Avg-acc using res1 was significantly higher than that using res2 and res3.

**AUC**

The AUC values of all the models were greater than 0.95 for all the operating conditions when using res1, and the AUC of the SpAE-OCSVM, the StAE-OCSVM, and the LSTM-OCSVM models were significantly lower when using res2 and res3.

**F1-score**

For the SpAE-OCSVM, StAE-OCSVM, and LSTM-OCSVM models, res1 obtained better results than the others. For the VAE-OCSVM model, res3 gave optimal results, but not as good as the other AE models.

It is easy to see that for the SpAE-OCSVM, the LSTM-OCSVM, and the StAE-OCSVM, res1 significantly outperformed the others in the Avg-acc, the AUC, and the F1-score. Taken together, res1 achieved better results than the other features when taken as a mapping feature and was more applicable to different AE models.

### 5.2. Comparison with AE and OCSVM

**Figure 8:**
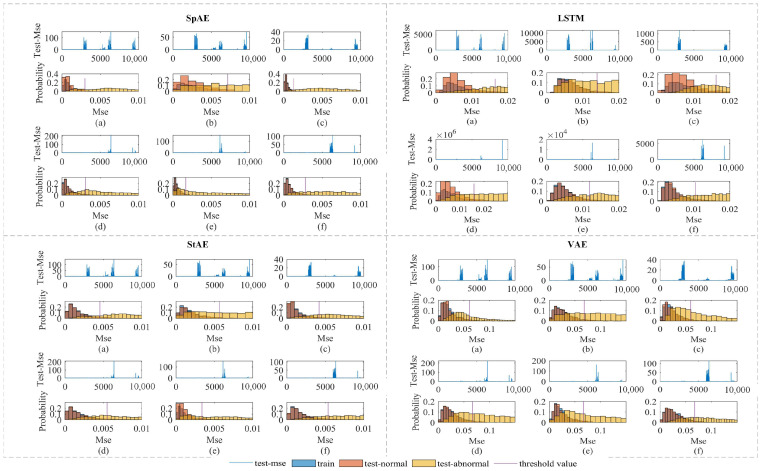
Part of the MSE of different models. (**a**–**f**) represents six different working conditions. The first row of images shows the MSE of each test data. The second row of images shows the probability distribution of the MSE. Blue represents the probability distribution of the training data, red represents the normal data in the test data, and yellow represents the abnormal data in the test data. The purple vertical line represents the threshold of the model.

Figure 8 shows the values of the reconstructed MSE for all the test data along with the MSE distribution for the training data, the normal test data, and part of the abnormal test data. We set the top 95% of the training data as the threshold. Regardless of which AE model was chosen, the MSE for most of the anomalous data were significantly larger than the normal data. However, there would still be some anomalous data whose MSE would be less than the threshold. This would result in a lower detection rate of anomalous data when using only AE for anomaly detection.

**Feature map**

**Figure 9:**
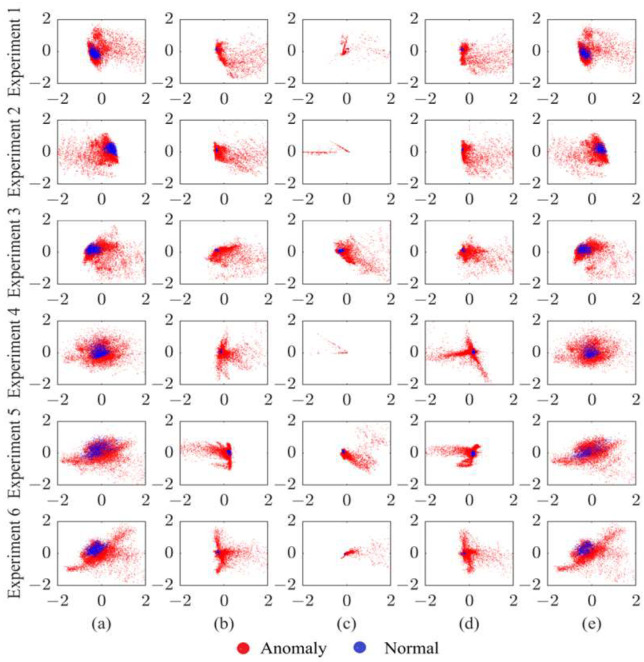
Features of different models after PCA. (**a**) original features; (**b**) SpAE-OCSVM; (**c**) LSTMAE-OCSVM; (**d**) StAE-OCSVM; (**e**) VAE-OCSVM.

Based on the calculations in Figure 8, we selected the res1 feature for comparison with the original feature. The 2D feature graph after the PCA of the test sample is in shown in Figure 9. The features after VAE mapping were not much different from the original feature distributions. With this structure and early stopping conditions, the VAE was less able to learn the harmonic reducer signal. This led to difficulties in responding to the differences between the normal and abnormal data with its mapping features. For the SpAE, the StAE, and the LSTM, the mapped health boundaries were more tightly constricted, resulting in higher detection rates of the anomalous data.

**Result**

**Figure 10:**
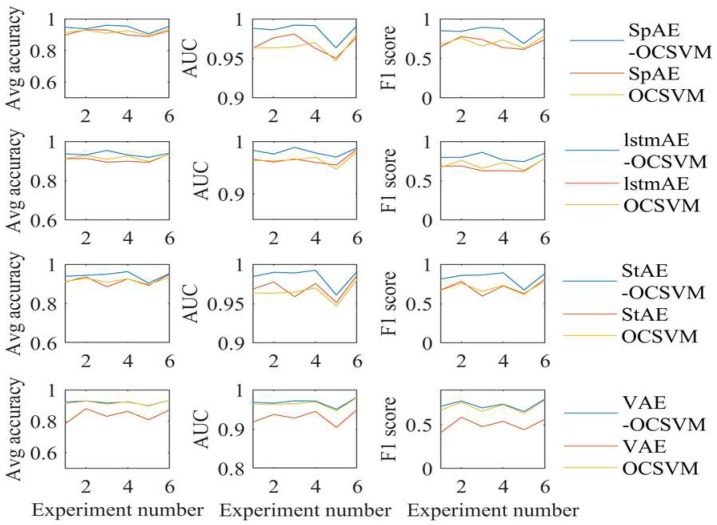
Results of AE-OCSVM, AE, and OCSVM.

We compared the computational results of the AE, OCSVM, and AE-OCSVM models [Figure 10, Table 3 and Table 4]. Overall, the results obtained from thr AE-OCSVM model were improved over either AE or OCSVM alone. For the SpAE-OCSVM, StAE-OCSVM, and LSTM-OCSVM, all the metrics (Avg-acc, AUC, and F1score) were all significantly higher than using AE or OCSVM alone. The VAE-OCSVM performed significantly better than the VAE alone, with a higher AUC and F1 score compared to the OCSVM alone. The best model was SpAE-OCSVM; the Avg-acc was higher than 90% in all the working conditions, and the overall Avg-acc was 94.25%, which was 2.47% higher than the OCSVM and 3.1% higher than the SpAE. The AUC was higher than the OCSVM by 0.0205, and the SpAE by 0.0172. The F1 score was higher than the OCSVM by 0.133, and the SpAE by 0.1472.

As shown in Table 3, the AE-OCSVM model sacrificed a portion of the accuracy of the normal data and improved the accuracy of the anomalous data more compared to using the AE or the OCSVM. In the case of the best-performing SpAE-OCSVM model, its average accuracy for normal data were 0.86% and 1.46%, lower than that of the SpAE and the OCSVM, respectively, but the average accuracy of anomaly detection was improved by 7.06% and 6.40%, respectively.

### 5.3. Comparison with Other Anomaly Detection Methods

**Figure 11:**
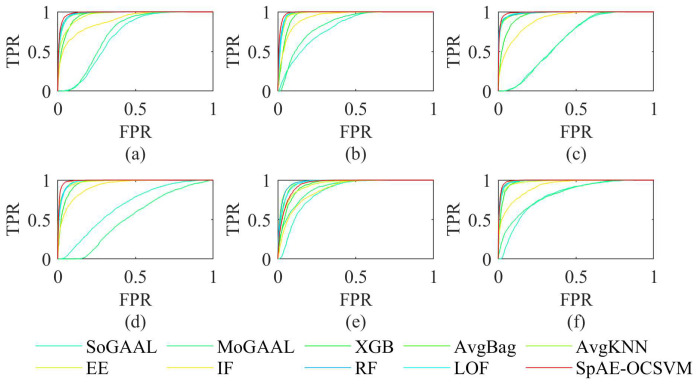
AUC of different methods.

We compared the SpAE-OCSVM model with other anomaly detection methods and the results obtained are shown in Table 5 and Table 6. The ROC curve is shown in Figure 11.

**ACC**

For normal data, the average accuracy of the SpAE-OCSVM was 1.37% lower than the AvgKNN and 1.13% lower than the RF for the six working conditions. However, the average anomaly detection accuracy of SpAE-OCSVM was higher than 95%, 2.86% higher than the AvgKNN, and 2.88% higher than the RF.

**AUC**

The SpAE-OCSVM had a minimum AUC of 0.9634 and an average AUC of 0.9854 for the six working conditions, which was at least 0.0041 higher than the other anomaly detection methods. The AUCs for all the working conditions were higher than 0.985, except for Experiment 5.

**F1-score**

The average F1-score of the SpAE-OCSVM for the six working conditions was 0.8379. It was at least 0.0705 higher than the other anomaly detection methods.

## 6. Conclusions

In this paper, we proposed a semi-supervised anomaly detection method for the harmonic reducers based on the AE-OCSVM model. The method was trained using only healthy data.

Traditional AEs uses an MSE as an anomaly detection threshold, which led to the lower detection rate of anomalous signals. To overcome this problem, we used an AE to map the original features to a new residual-based feature space and further determined whether the signal was anomalous or not by using an OCSVM model trained on healthy signals. This removed the non-discriminative part of the original features, while retaining more details of the differences compared to a single MSE.

The results of the six sets of harmonic reducer experiments under different operating conditions showed that the AE-OCSVM model had better performance in the Avg-acc, the AUC, and the F1-score than using the AE or the OCSVM alone. Especially for anomalous data, the AE-OCSVM model has a higher detection rate.

After comparing various residual-based features, we found that using the residual directly as “difference features” gave the best results. After comparing multiple AE-OCSVM models, the SpAE-OCSVM had the best performance. Compared to the other anomaly detection methods, the SpAE-OCSVM still had excellent anomaly detection performance.

In future work, we will make improvements in feature extraction, drawing on relevant expert knowledge, and extracting features that are more highly associated with anomalies to improve the accuracy and interpretability of the method. At the same time, we will also improve the generalization performance of the model and expect to achieve anomaly detection for all the operating conditions of the harmonic reducers using only one model.

## Figures and Tables

**Table 1:** Feature extraction.

Time Domain					Frequency Domain
$X_{1}=\max(x_{i})$	$X_{4}=X_{1}-X_{2}$	$X_{7}=\frac{1}{N-1}\sum_{i=1}^{N}{\left(x_{i}-X_{3}\right)}^{2}$	$X_{10}=\frac{1}{{X_{8}}^{4}}\frac{1}{N}\sum_{i=1}^{N}{\left(x_{i}-X_{3}\right)}^{4}$	$X_{13}=X_{9}/X_{6}$	$X_{16}=\frac{1}{N}\sum_{i=1}^{N}f_{i}$
$X_{2}=\min(x_{i})$	$X_{5}=\frac{1}{N}\sum_{i=1}^{N}\left|x_{i}\right|$	$X_{8}=\sqrt{X_{7}}$	$X_{11}=\frac{1}{{X_{8}}^{3}}\frac{1}{N}\sum_{i=1}^{N}{\left(x_{i}-X_{3}\right)}^{3}$	$X_{14}=X_{9}/X_{5}$	$X_{17}=\sqrt{\frac{1}{N}\sum_{i=1}^{N}{f_{i}}^{2}}$
$X_{3}=\frac{1}{N}\sum_{i=1}^{N}x_{i}$	$X_{6}=\sqrt{\frac{\sum_{i=1}^{N}{x_{i}}^{2}}{N}}$	$X_{9}=\max(\left|x_{i}\right|)$	$X_{12}=X_{6}/X_{5}$	$X_{15}=\frac{X_{9}}{(\frac{1}{N}\sum_{i=1}^{N}\sqrt{\left|x_{i}\right|}{)}^{2}}$	$X_{18}=\sqrt{\frac{\sum_{i=1}^{N}{\left(f_{i}-X_{16}\right)}^{2}}{N}}$

**Table 2:** Experiment condition (sampling frequency: 100 kHz; data length: 30,000; training sample: 600 of normal data; testing sample: 400 of each type of fault).

Experiment Number	Load (Nm)	Speed (rpm)	Fault Type
1	−10, 0, 10	600	Normal; F1; F2; F3; F4; F5; F6; F7
2	−10, 0, 10	800	
3	−10, 0, 10	1000	
4	−10, 0, 10	1600	
5	−10, 0, 10	1800	
6	−10, 0, 10	2000	

**Table 3:** Accuracy of normal and abnormal data (N: normal A: abnormal).

Experiment	1		2		3		4		5		6	
	N	A	N	A	N	A	N	A	N	A	N	A
VAE	0.9558	0.6123	0.9400	0.8199	0.9533	0.7098	0.9525	0.7745	0.9558	0.6637	0.9467	0.7989
OCSVM	0.9533	0.8712	0.9333	0.9246	0.9542	0.8635	0.9408	0.9099	0.9442	0.8500	0.9325	0.9365
VAE-OCSVM	0.9467	0.8977	0.9267	0.9323	0.9433	0.8889	0.9317	0.9148	0.9300	0.8675	0.9258	0.9429
LSTMAE	0.9400	0.8840	0.9400	0.8864	0.9400	0.8479	0.9542	0.8438	0.9425	0.8438	0.9350	0.9365
OCSVM	0.9533	0.8712	0.9333	0.9246	0.9542	0.8635	0.9408	0.9099	0.9442	0.8500	0.9325	0.9365
LSTMAE-OCSVM	0.9317	0.9419	0.9183	0.9444	0.9433	0.9644	0.9358	0.9257	0.9167	0.9212	0.9125	0.9667
StAE	0.9433	0.8764	0.9383	0.9340	0.9475	0.8231	0.9458	0.9051	0.9425	0.8436	0.9558	0.9426
OCSVM	0.9533	0.8712	0.9333	0.9246	0.9542	0.8635	0.9408	0.9099	0.9442	0.8500	0.9325	0.9365
StAE-OCSVM	0.9292	0.9488	0.9200	0.9682	0.9308	0.9680	0.9508	0.9742	0.9258	0.8819	0.9317	0.9751
SpAE	0.9325	0.8617	0.9283	0.9337	0.9483	0.9118	0.9408	0.8555	0.9350	0.8406	0.9375	0.9126
OCSVM	0.9533	0.8712	0.9333	0.9246	0.9542	0.8635	0.9408	0.9099	0.9442	0.8500	0.9325	0.9365
SpAE-OCSVM	0.9325	**0.9627**	0.9100	**0.9637**	0.9425	**0.9761**	0.9367	**0.9712**	0.9183	**0.8924**	0.9308	**0.9733**

**Table 4:** Indicators of different models.

Experiment	1	2	3	4	5	6	Avg
Avg-acc							
VAE	0.7840	0.8799	0.8315	0.8635	0.8098	0.8728	0.8403
LSTMAE	0.9120	0.9132	0.8939	0.8990	0.8932	0.9358	0.9078
StAE	0.9099	0.9362	0.8853	0.9255	0.8930	0.9492	0.9165
SpAE	0.8971	0.9310	0.9301	0.8982	0.8878	0.9251	0.9115
OCSVM	0.9123	0.9290	0.9088	0.9254	0.8971	0.9345	0.9178
VAE+OCSVM	0.9222	0.9295	0.9161	0.9232	0.8988	0.9343	0.9207
LSTMAE+OCSVM	0.9368	0.9314	0.9539	0.9308	0.9189	0.9396	0.9352
StAE+OCSVM	0.9390	0.9441	0.9494	0.9625	0.9039	0.9534	0.9420
SpAE+OCSVM	**0.9476**	0.9368	**0.9593**	0.9539	**0.9054**	0.9521	**0.9425**
AUC							
VAE	0.9180	0.9373	0.9281	0.9452	0.9046	0.9500	0.9305
LSTMAE	0.9659	0.9605	0.9663	0.9599	0.9549	0.9856	0.9655
StAE	0.9684	0.9775	0.9588	0.9756	0.9513	0.9864	0.9697
SpAE	0.9627	0.9757	0.9808	0.9628	0.9501	0.9769	0.9682
OCSVM	0.9636	0.9632	0.9648	0.9699	0.9469	0.9809	0.9649
VAE+OCSVM	0.9683	0.9661	0.9720	0.9715	0.9506	0.9818	0.9684
LSTMAE+OCSVM	0.9829	0.9759	0.9886	0.9779	0.9699	0.9880	0.9805
StAE+OCSVM	0.9844	0.9898	0.9890	0.9923	0.9609	0.9912	0.9846
SpAE+OCSVM	**0.9881**	0.9861	**0.9921**	0.9913	**0.9634**	0.9912	**0.9854**
F1-score							
VAE	0.4094	0.5873	0.4785	0.5395	0.4435	0.5645	0.5038
LSTMAE	0.6832	0.6874	0.6256	0.6262	0.6209	0.7860	0.6716
StAE	0.6718	0.7819	0.5948	0.7248	0.6206	0.8109	0.7008
SpAE	0.6429	0.7760	0.7392	0.6373	0.6129	0.7355	0.6907
OCSVM	0.6678	0.7586	0.6558	0.7317	0.6307	0.7847	0.7049
VAE+OCSVM	0.7111	0.7720	0.6934	0.7370	0.6509	0.7961	0.7268
LSTMAE+OCSVM	0.7969	0.7960	0.8605	0.7621	0.7427	0.8505	0.8014
StAE+OCSVM	0.8124	0.8588	0.8639	0.8921	0.6727	0.8848	0.8308
SpAE+OCSVM	**0.8503**	0.8410	**0.8934**	0.8761	**0.6875**	0.8792	**0.8379**

**Table 5:** Acc of different methods.

	Normal						Abnormal						Avg
	1	2	3	4	5	6	1	2	3	4	5	6	
SoGAAL	0.8925	0.8842	0.9058	0.9108	0.8917	0.8983	0.1449	0.0271	0.1392	0.0827	0.0386	0.0407	0.4727
MoGAAL	0.8950	0.8825	0.9117	0.9033	0.9042	0.9008	0.1389	0.0398	0.1402	0.2192	0.0030	0.0089	0.4807
XGB	0.6475	0.7258	0.6308	0.5533	0.6217	0.5858	0.9575	0.9435	0.9618	0.9743	0.9437	0.9825	0.5816
IF	0.8675	0.8400	0.8650	0.8667	0.8467	0.8242	0.6882	0.8824	0.7765	0.8258	0.7479	0.8331	0.8384
LOF	0.9967	0.9958	0.9933	0.9967	0.9983	0.9925	0.7957	0.8854	0.7867	0.7915	0.6869	0.8492	0.8987
EE	0.9117	0.8700	0.8883	0.9083	0.8692	0.9033	0.8617	0.9512	0.9614	0.9367	0.8644	0.9446	0.9062
AvgBag	0.9775	0.9183	0.8883	0.8875	0.9258	0.9508	0.8932	0.9529	0.9762	0.9517	0.9224	0.9483	0.9322
RF	0.9467	0.9342	0.9542	0.9492	0.9483	0.9292	0.9173	0.9481	0.9243	0.9230	0.8818	0.9530	0.9333
AvgKNN	0.9508	0.9383	0.9508	0.9575	0.9375	0.9408	0.9092	0.9442	0.9114	0.9140	0.9119	0.9498	0.9356
SpAE-OCSVM	0.9283	0.9217	0.9433	0.9383	0.9325	0.9292	**0.9582**	**0.9657**	**0.9796**	**0.9742**	**0.8786**	**0.9760**	**0.9425**

**Table 6:** AUC and F1-score of different methods.

	AUC							F1						
	1	2	3	4	5	6	Avg	1	2	3	4	5	6	Avg
SoGAAL	0.7951	0.7922	0.6489	0.7617	0.7650	0.8152	0.7630	0.2085	0.2011	0.2262	0.2082	0.2078	0.2060	0.2096
MoGAAL	0.7554	0.8314	0.6593	0.6931	0.8207	0.8321	0.7653	0.2097	0.2041	0.2263	0.2232	0.2077	0.2058	0.2128
XGB	0.9587	0.9586	0.9575	0.9629	0.9396	0.9764	0.9589	0.3324	0.2813	0.2317	0.2819	0.2821	0.2627	0.2787
IF	0.9119	0.9399	0.9237	0.9426	0.8971	0.9429	0.9264	0.4610	0.6546	0.5358	0.6045	0.4840	0.6105	0.5584
LOF	0.9795	0.9789	0.9856	0.9795	0.9634	0.9852	0.9787	0.5855	0.7135	0.5736	0.5806	0.4775	0.6553	0.5977
EE	0.9587	0.9754	0.9836	0.9790	0.9414	0.9787	0.9695	0.6337	0.7885	0.8235	0.7750	0.6184	0.7889	0.7380
AvgBag	0.9784	0.9785	0.9859	0.9793	0.9613	0.9853	0.9781	0.7405	0.8069	0.7670	0.7581	0.6536	0.8250	0.7585
RF	0.9821	0.9823	0.9862	0.9818	0.9743	0.9878	0.9824	0.6885	0.8163	0.8690	0.7772	**0.7662**	0.8383	0.7926
AvgKNN	0.9786	0.9812	0.9832	0.9814	**0.9748**	0.9885	0.9813	0.7388	0.8059	0.7486	0.7527	0.7359	0.8225	0.7674
SpAE-OCSVM	**0.9881**	**0.9861**	**0.9921**	**0.9913**	0.9634	**0.9912**	**0.9854**	**0.8503**	**0.8410**	**0.8934**	**0.8761**	0.6875	**0.8792**	**0.8379**

## Data Availability

The data presented in this study are available on request from the corresponding author due to limitations.
